# Safety of Shujinjianyao pill in clinical real world: A prospective, observational, multicenter, large-sample study Protocol

**DOI:** 10.1097/MD.0000000000016853

**Published:** 2019-08-16

**Authors:** Xin Cui, Long Liang, Lianxin Wang, Zhifei Wang, Yuanyuan Li, Yang Gao, Cheng Zhang, Menghua Sun, Shiheng Wang, Jiani Liu, Yue Zhang, Zhibo wang, Xu Wei, Yanming Xie

**Affiliations:** a Institute of Basic Research in Clinical Medicine; b Wangjing Hospital; c China Institute for History of Medicine and Medical Literature; d Xiyuan Hospital, China Academy of Chinese Medical Sciences, Beijing; e Longhua Hospital, Shanghai University of Traditional Chinese Medicine, Shanghai, China.

**Keywords:** observational study, protocol, safety, shujinjianyao pill, the real world

## Abstract

**Background:**

Low back pain is a common health problem worldwide, which also is a leading cause of long-term disability and has an important effect on the global economy and society. Usually, conservative therapies are used to treat low back pain. As a kind of Chinese patent medicine, Shujinjianyao pill (SJJYP) has a great curative effect on low back pain. However, its safety has not been studied yet. Therefore, we carried out this clinical trial to observe the safety of SJJYP in the real world.

**Methods:**

First, participants need to meet the medication standards according to inclusion and exclusion criteria. Then, participants are conducted safety examination before taking SJJYP. After qualified screening, participants can be enrolled into the group. Second, all enrolled participants will receive SJJJYP for a period of 4 weeks. During the observation period, participants need to return to the hospital for a subsequent visit after 2 weeks of medication, and come to the hospital for safety check after 4 weeks of medication. Third, telephone follow-up is used to investigate any participants’ physical discomfort after 6 to 8 weeks (2–4 weeks after medication withdrawal). After all these steps are completed, clinical observation is finished. If any adverse events occur during this process, we will record them in time. When serious adverse events occur, we will use nested case–control study to explore the causes and mechanisms.

**Discussion:**

This study will obtain the safety results of SJJYP in clinical real world, which will offer a scientific basis for clinicians in the treatment of low back pain, and also provide a methodological basis for the safety study of other medicines.

**Trial registration:**

ClinicalTrial.gov registration number is NCT03598153. This study was approved by the ethics committee of Wangjing hospital, China Academy of Chinese Medical Sciences (WJEC-KT-2018-012-P002).

## 1 Introduction

Low back pain is a very common health problem.^[1,2,3,4]^ At least 80% of people have suffered from low back pain at some point in their lives.^[5,6,7,8]^ The lifetime incidence of low back pain is 58% to 84%.^[8,9,10]^ The prevalence rate is slightly higher in women than in men, and the recurrence rate is also relatively higher.^[8,10]^ Risk factors for low back pain include obesity, age, heavy physical work, smoking, psychosocial factors (such as depression, stress, among others), and so on.^[11]^ According to a recent global survey, low back pain is the leading cause of long-term disability.^[12]^ The social and economic burden regarding low back pain are enormous challenges all over the world, which is a major cause of global absenteeism.^[13]^ In the United States, the direct cost of lower back pain was estimated at $34 billion annually. But if lost wages and other indirect costs were taken into account, it could exceed $100 billion.^[8,14]^

Most low back pain is nonneuropathic, which is confined to the spine and paravertebral region and has no neurological symptoms.^[15]^ Nerve root involvement (reported as sciatica) is rare.^[16]^ For the treatment of low back pain, doctors always rely on conservative treatment with a combination of physical therapy, exercise programs, analgesics, anti-inflammatory drugs, and opioids, unless the patients with low back pain or sciatica have “dangerous signals” of malignant tumors, infections, fractures, or cauda equina syndrome.^[17,18,19,20,21]^ As a treasure of China, TCM has unique advantages in the treatment of some chronic complex diseases.^[22,23,24]^ Shujinjianyao pill (SJJYP) is a kind of Chinese patent medicine containing many kinds of traditional Chinese herbs, which is mainly used to treat low back pain, sciatica, and other diseases in clinic.^[25,26,27]^ However, there is no study that has been conducted on its safety. Therefore, we will carry out a multicenter and large-sample clinical monitoring to observe the safety of this drug, which can provide scientific basis for clinical medication in the future.

## 2 Methods

### 2.1 Study aims

On the one hand, the purpose of this clinical trial is to obtain the safety of SJJYP in the real world. On the other hand, the types, incidence rates, characteristics, and degrees of adverse reactions (ADRs) of SJJYP were obtained, and the influencing factors were explored.

### 2.2 Design/setting

This clinical trial is designed as a observational, prospective, multi-center, large-sample study. All participants will be recruited from the following 25 hospitals: Wangjing Hospital of China Academy of Chinese Medical Sciences (CACMS), Xiyuan Hospital of CACMS, Affiliated Hospital of Traditional Chinese Medicine (TCM) of Southwest Medical University, Beijing University of Chinese Medicine Third Affiliated Hospital, Affiliated Hospital of Changchun University of Chinese Medicine, Affiliated Hospital of Shandong University of TCM, Gansu Provincial Hospital of TCM, The First Hospital of Hunan University of Chinese Medicine, Shunde Hospital of Guangzhou University of Chinese Medicine, Beijing Hepingli Hospital, Beijing Changping Hospital of TCM, Beijing Yanqing Hospital of TCM, Beijing Mentougou Hospital of TCM, Beijing Gulou Hospital of TCM, Beijing Shunyi hospital of TCM, Baiyin Branch of Gansu Hospital of TCM, Dunhuang Hospital of TCM, Neijiang Hospital of TCM, Jiangan Hospital of TCM, Liangshan Prefecture Integrated Traditional Chinese and Western Medicine Hospital, Wuhan hospital of TCM, Xi’an Honghui hospital, China-Japan Friendship Hospital, and Mianyang Hospital of TCM, Fushun Hospital of TCM.

**Figure 1:**
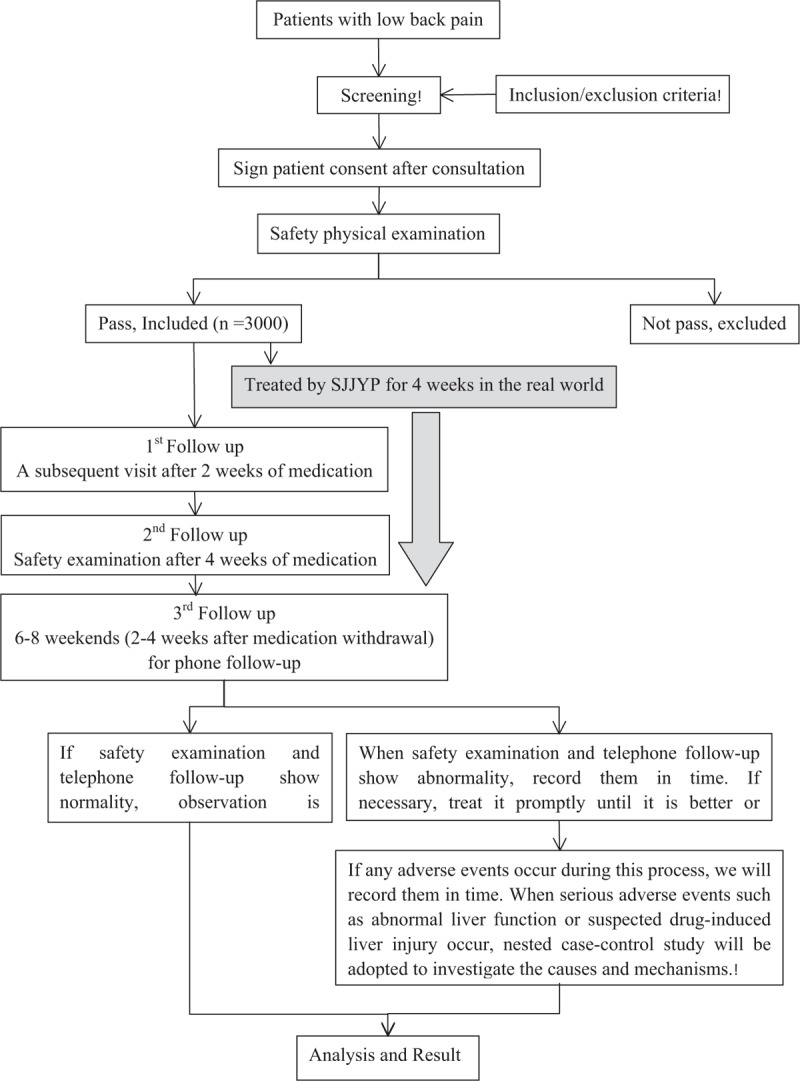
Flow diagram of the clinical trial.

Participants are recruited mainly through recruitment advertisements in hospitals mentioned. Enrollment needs to be determined according to the inclusion and exclusion criteria. At the same time, the participants voluntarily sign the patient consent and need to pass the relevant safety examination. After that, they can be officially enrolled (detailed procedures for this clinical trial are shown in Fig. 1). According to National Medical Products Administration asked for opinions on *the notice on promoting key drug monitoring work of manufacturers (draft for opinions)* ,^[28]^ 3000 participants are expected to be enrolled in this study, who will receive free 4-week treatment of SJJYP. Meanwhile, the physical status of participants is followed up after medication for 2 weeks, the safety examination is reviewed after medication for 4 (±3 days), and 6 to 8 weekends (2–4 weeks after medication withdrawal) for phone follow-up. All participants enrolled in this clinical trial are covered by insurance.

### 2.3 Study registration

This clinical trial has been registered on the international Clinical Trials registration platform (ClinicalTrials. gov), and the registration number is NCT03598153.

### 2.4 Eligibility criteria

#### 2.4.1 Inclusion criteria

1. Patients with normal liver function before medication.
2. Patients suffered from low back pain, knee pain, or sciatica.
3. Patients aged between 18 and 80 years.

#### 2.4.2 Exclusion Criteria

1. Patients who are pregnant, phrenetic, and suffered from serious illness.
2. Patients suffered from cirrhosis or liver cancer or various kinds of hepatitis, such as viral hepatitis, autoimmune hepatitis, alcoholic hepatitis, and so on.
3. Safety examination showed that the alanine aminotransferase (ALT) and aspartate aminotransferase (AST) values are 120% higher than the maximum normal values.

### 2.5 Intervention

SJJYP was given to treat all eligible patients. The medication period is 4 weeks (±3 days). According to the instruction, the frequency and dosage of SJJYP can be reasonably used. The combined drugs and other treatments are not considered during the whole process of monitoring. To improve adherence of participants, the rest of the drugs and the bottle needs to be returned to the monitoring center after the monitoring.

### 2.6 Outcome measures

#### 2.6.1 Primary outcome

The primary outcome of this study is liver function test, which mainly includes ALT, AST, gamma-glutamyl transpeptidase, and total bilirubin (TBil). Liver function tests are performed before and 1 month after treatment.

#### 2.6.2 Secondary outcome

1. Kidney function test: Apart from liver function test, kidney function test is the secondary outcome including creatinine (Cr) and blood urea nitrogen (BUN).
2. Blood routine examination: Blood routine is the examination of blood condition and disease by observing the number and distribution of blood cells. At the same time, many indicators are sensitive to pathological changes. Hence, blood routine is regarded as a basic test to observe the safety of SJJYP.
3. Urine routine examination: Urinary routine plays a vital role in medical examination. Proteinuria or constituents in urinary sediment can occur in many early renal diseases. Concurrently, it also has important reference value for the diagnosis of some diseases such as diabetes, blood diseases, liver or gallbladder diseases, epidemic hemorrhagic fever, and so on.
4. Electrocardiogram (ECG): ECG is the most common method to detect myocardial ischemia and diagnose angina pectoris, which can contribute to reflect on the safety of SJJYP.

### 2.7 Adverse events

If any adverse events occur in the process of monitoring, such as suspected drug-induced liver injury, allergic reactions, and digestive tract symptoms, patients first need to stop taking drugs, and further observation is also needed. In addition, as for patients with drug-related adverse events, the patient consent for additional blood tests is signed after consulting. Then, their blood are collected and stored in a refrigerator at −80°C, which is transported to the laboratory regularly for unified testing. Blood are only used for clinical assessment and investigating causes and mechanism of adverse events. All adverse events will be followed up until they are properly resolved or the patient is in stable condition. Referring to national regulations and Good Clinical Practice (GCP) regulations, ADR monitoring centers are reported level by level according to the degree of severity and type of adverse events.

### 2.8 Composition of monitoring management

The monitoring management of this study is composed of academic expert committee, data and safety inspection expert committee and terminal event interpretation expert committee, which provide strong guarantee for its advancement, rationality, and operability.

Regular ADRs/adverse events case seminars are held to evaluate and analyze the occurrence and causality of ADRs/adverse events. Each center needs to establish an Adverse Drug Reaction/Event Monitoring Expert Committee, which is responsible for monitoring related affairs of the center.

### 2.9 Data collection and management

The monitors of 25 monitoring centers collect personal information of participants, medication, and follow-up information. When inputting observation data, they use the form of “double entry.” Meanwhile, we invite third-party outside the project team to control the quality of the entire monitoring process, which is responsible for verifying the authenticity, completeness, timeliness, standardization of data.

Data will be processed anonymously, omitting the information that can identify the participant's individual identity. Strict safety and confidentiality measures will be established in the archives of clinical trial institutions.

### 2.10 Statistical analysis

Statistical analysis is undertaken by professional medical statisticians. At the same time, they also participate in the whole process from study design, implementation to analysis. Data format may be modified during the process if necessary. Accordingly, statistical analysis report will be provided after the analysis completed.

### 2.11 Ethics and dissemination

This clinical trial has been approved by the ethics committee of Wangjing Hospital of CACMS (No.: WJEC-KT-2018-012-P002).

To protect privacy of participants, only those who participate in monitoring and inspectors have access to patients’ personal medical records. They will keep all information of patients confidential.

The ethics committee and the drug regulatory department have the right to inspect the records of clinical trials.

### 2.12 Trial status

The clinical trial is still recruiting participants, which is expected to be completed in October 2019.

## 3 Discussion

In this clinical trial, the safety of SJJYP in the real world is mainly assessed by observing the changes of liver function, renal function, blood routine, urine routine and ECG before and after taking SJJYP. Absolutely, follow-up is also very important and essential.

This trial has been registered on ClinicalTrials.gov, which will make the procedure of study more transparent and credible. In the meantime, 25 clinical centers are distributed all over China, which can effectively reduce the regional bias. Simultaneously, 3000 large samples will be recruited in this study according to relevant standards, which will make the evidence more convincing. In the event of suspected drug-induced liver injury or abnormal liver function or other serious drug-related adverse events, nested case–control study will be used to investigate the causes and mechanisms.

We hoped that more reliable research evidence can be obtained through this study, which are conducive to provide a better scientific basis for clinicians to rationally choose medication in the treatment of low back pain.

## Author contributions

**Conceptualization:** Xin Cui, Long Liang.

**Data curation:** Yuanyuan Li, Jiani Liu, Yue Zhang.

**Methodology:** Xu Wei, Yanming Xie.

**Resources:** Zhifei Wang, Cheng Zhang, Menghua Sun.

**Software:** Yang Gao, Shiheng Wang, Zhibo Wang.

**Supervision:** Yanming Xie.

**Writing – original draft:** Xin Cui, Long Liang, Lianxin Wang.

**Writing – review & editing:** Xin Cui, Long Liang, Xu Wei, Yanming Xie, Lianxin Wang, Zhifei Wang, Yuanyuan Li.

Xin Cui orcid: 0000-0003-2257-6724.
